# Glycyrrhetinic Acid Liposomes Containing Mannose-Diester Lauric Diacid-Cholesterol Conjugate Synthesized by Lipase-Catalytic Acylation for Liver-Specific Delivery

**DOI:** 10.3390/molecules22101598

**Published:** 2017-09-24

**Authors:** Jing Chen, Yuchao Chen, Yi Cheng, Youheng Gao

**Affiliations:** 1 Shool of Chinese Materia Medica, Guangzhou University of Chinese Medicine, Guangzhou 510006, China; 13699738965@163.com; 2 The Second Affiliated Hospital of Guangzhou University of Chinese Medicine, Guangzhou 510115, China; lcnacj@126.com; 3 Section of Immunology, Guangdong Provincial Academy of Chinese Medical Sciences, Guangzhou 510006, China; 4 Postdoctoral Programme, Guangzhou University of Chinese Medicine, Guangzhou 510006, China

**Keywords:** lipase-catalytic acylation, mannose-diester lauric diacid-cholesterol, glycyrrhetinic acid liposomes, mannose receptor, liver-targeting

## Abstract

Mannose-diester lauric diacid-cholesterol (Man-DLD-Chol), as a liposomal target ligand, was synthesized by lipase catalyzed in a non-aqueous medium. Its chemical structure was confirmed by mass spectrometry (MS) and nuclear magnetic resonance (NMR) spectroscopy. Glycyrrhetinic acid (GA) liposomes containing Man-DLD-Chol (Man-DLD-Chol-GA-Lp) were prepared by the film-dispersion method. We evaluated the characterizations of liposomes, drug-release in vitro, the hemolytic test, cellular uptake, pharmacokinetics, and the tissue distributions. The cellular uptake in vitro suggested that the uptake of Man-DLD-Chol-modified liposomes was significantly higher than that of unmodified liposomes in HepG2 cells. Pharmacokinetic parameters indicated that Man-DLD-Chol-GA-Lp was eliminated more rapidly than GA-Lp. In tissue distributions, the targeting efficiency (Te) of Man-DLD-Chol-GA-Lp on liver was 54.67%, relative targeting efficiency (R_Te_) was 3.39, relative uptake rate (Re) was 4.78, and peak concentration ratio (Ce) was 3.46. All these results supported the hypothesis that Man-DLD-Chol would be an efficient liposomal carrier, and demonstrated that Man-DLD-Chol-GA-Lp has potential as a drug delivery for liver-targeting therapy.

## 1. Introduction

Liver diseases can be caused by various factors that damage the liver. There are four major liver diseases, including fatter liver, cirrhosis, hepatitis, and hepatocellular carcinoma, while the latter two belong to serious public health issues [1,2,3]. According to 2015 cancer statistics, the incidence of hepatocellular carcinoma was fourth in all cancers and its mortality rate was the third highest in China [4]. However, most conventional anti-tumor drugs for treatment of hepatocellular carcinoma have little or no specificity, which results in systemic toxicity, causing undesirable side effects [5]. Therefore, a drug delivery system targeting hepatic cells would be an attractive approach to cure hepatic diseases.

18β-Glycyrrhetinic acid (GA), serving as an aglycone and active metabolite of glycyrrhizic acid, shows a variety of pharmacological effects, such as a potential anti-hepatotoxic agent [6], hepatoprotective effect [7,8], inhibition of hepatocellular carcinoma [9,10], anti-inflammation [11], and anti-viral [12] and interferon induction [13]. These findings suggested that GA may become a suitable candidate for the clinical treatment of hepatic diseases. However, GA is a lipophilic drug with a very low solubility in water, which may result in poor bioavailability. In addition, GA may cause unwanted sodium retention and potassium loss [14]. A liposomal drug delivery system is a suitable strategy to avoid the side effects and maintain an effective concentration of the drug [15]. Liposomes are considered to be a mainstream drug delivery technology due to the relative stability in the blood, the high cell affinity, and tissue compatibility without immunosuppressive effects to the human body [16,17].

Receptor-mediated delivery is a favorable method employing novel carriers to insert the drug into specific targeted tissues or cells [18]. Some literatures reported that PEG-modified liposome loading GA, and a review on GA receptor and GA-delivering carriers [19,20,21]. However, this study was novel because it provided a new kind of mannosylated conjugate for liposomal modification that resulted in better liver-targeting of GA. It was reported that mannose receptors (MR) are highly expressed on the liver endothelial cells and dendritic cells [22,23]. The carbohydrate-recognition domains of MR have the ability to recognize compounds containing terminal mannose, fucose or *N*-acetyl-glucosamine residues [24,25,26]. This mechanism would be a promising approach to achieve target specificity of liver endothelial cells. Liposomes have good cell affinity and biocompatibility and the capacity for surface modification [27,28,29]. The surface modification of liposomes could also decorate with various targeting ligands to increase the amount of drug delivery and reduce side effects. Thus, using modified liposomes with ligands for receptors on hepatic cells is considered to be an efficient method for the targeted delivery of drugs.

The ability of mannose receptor to recognize mannosylated liposomes depends on three factors: a hydrophilic mannosylated group with high affinity for hepatic mannose receptor on the liposomal surface [30], a hydrophobic part enhancing the stability of incorporation in the lipid bilayer of the liposomes and reducing untimely exchange to other lipid compartments [31,32], a spacer length between the mannose moiety and the liposome [33,34], and several mannosylated cholesterol conjugates synthesized successfully have been reported [35,36,37]. However, these mannosylated cholesterol derivatives were synthesized by conventional chemical methods. The applications could be somewhat hampered due to the multiple synthesis steps, complicated conditions, the tedious product isolation, and the environmental concern of the process [38]. Meanwhile, the enzymatic synthesis has distinct advantages, such as mild reaction conditions, high regioselectivity, desirable productivity and environmental benign [39,40,41]. Thus, we chose the method to achieve enzymatic synthesis of mannose-dister lauric diacid-cholesterol (Man-DLD-Chol) in non-aqueous medium.

We designed Man-DLD-Chol byenzymatic acylation in organicmedia. The Man-DLD-Chol consisted of three parts: mannose, diester lauric diacid (DLD), and cholesterol. Mannose, a hydrophilic part, was regarded as a recognition moiety to MR in hepatocytes. Cholesterol, one of the components of liposomes, was embedded into the lipid bilayer of liposomes to improve the stability of the drug delivery system [42]. DLD, a spacer part of the linker between cholesterol and mannose, was chosen to ensure an efficient exposure of the mannose function and a tight association of the liposomes with the mannose receptor.

This was the first time our experimental team to synthesize the Man-DLD-Chol conjugate and used Man-DLD-Chol-modified GA liposomes. In this study, Man-DLD-Chol was synthesized under the lipase-catalyst. The 3-hydroxyl group in cholesterol had the advantage of acylation, forming a stable structure with the P-π conjugated system, and while DLD was excessive, the diester compound could be inhibited. Therefore, the enzymatic synthesis with high substrate conversion and high regioselectivity was reacted successfully. The chemical structures of Man-DLD-Chol were confirmed by mass spectrometry (MS) and nuclear magnetic resonance (NMR) spectroscopy. We aimed to confirm whether the GA liposome containing Man-DLD-Chol has liver-targeting efficiency or not. We prepared conventional GA liposomes (GA-Lp) and GA-Lp containing Man-DLD-Chol (Man-DLD-Chol-GA-Lp). GA-Lp and Man-DLD-Chol-GA-Lp were evaluated, including the preparation, characterizations, GA release in vitro, hemolytic test, and cellular uptake in vitro. We deeply investigated the pharmacokinetics and tissue distributions of Man-DLD-Chol-GA-Lp, GA-Lp, and GA solution (GA-S) by intravenous injection. The pharmacokinetic parameters and bio-distribution data were calculated. The detection method of high-performance liquid chromatography-tandem mass spectrometry (LC-MS/MS) was more sensitive, selective, and accurate in comparison with detecting techniques. The LC-MS/MS method for detection of GA in plasma and tissues was rarely reported. The in vitro and in vivo evaluations provided important evidences for the further clinical development of Man-DLD-Chol-GA-Lp. These results would favor the hypothesis that GA liposomes containing targeted ligands of Man-DLD-Chol enhanced the liver-targeting efficiency.

## 2. Results and Discussion

### 2.1. Synthesis of Man-DLD-Chol

The synthesis of Man-DLD-Chol was a lipase-catalyzed reaction in a non-aqueous phase. Both enzymatic synthesis steps generated vinyl alcohol, which could turn into acetaldehyde. The acetaldehyde was easily volatile because of its low boiling point. Thus, the reactions were irreversible and complete. DLD-cholesterol (DLD-Chol) and Man-DLD-Chol were synthesized successfully by enzymatic acylation in organic media.

The molecular weight detected was 305.17 [M + Na]^+^ by ESI-MS (positive-ion mode), which indicated that the identical product of DLD (molecular weight: 282.17) was obtained. In the ^13^C NMR spectrum of the product showed that the chemical shift values of vinyl ester group at C1 and C2 atom were 98.3 ppm and 141.7 ppm, the carbonyl groups at the C3 atom were shifted to a higher magnetic field about δ 3.6 ppm (from δ 174.4 ppm to δ 170.8 ppm), which indicated esterification proceeded between vinyl acetate and laurel acid.

The molecular weight detected was 647.50 [M + Na]^+^ in positive-ion mode, which suggested that the identical intermediate of DLD-Chol (molecular weight: 624.50) was synthesized successfully. There are two carbonyl groups in DLD-Chol, the chemical shift values were 173.1 ppm and δ 170.7 ppm. The chemical shift of acylation at a hydroxyl group has been reported to show a downfield shift in ^13^C NMR [43]. The chemical shift value at the C3 atom was shifted to a lower magnetic field (δ 73.5 ppm), about a 2 ppm shift. Meanwhile, neighboring carbon atoms (C2 and C4) were shifted to a higher magnetic field, about 2.8 ppm (from δ 31.7 ppm to δ 28.9 ppm) and a 2.8 ppm ( δ 42.4 ppm to δ 39.6 ppm), indicating that the acylation proceeded between the hydroxyl group in cholesterol and DLD.

The molecular weight detected was around 783.54 [M + Na]^+^ in positive-ion mode, which proved that the product obtained was identical with Man-DLD-Chol (molecular weight: 760.54). According to some published literature, the acylation of a hydroxyl group of sugar had been reported to show a downfield shift of the peak corresponding to O-acylated carbon atom and a higher magnetic shift of the peak corresponding to a neighboring carbon atom [44,45]. The product of the Man-DLD-Chol structure was determined by ^13^C NMR at 100 MHz. The methylene group at the C-6″ atom was shifted to a lower magnetic field, about 2.1 ppm (from δ 63.4 ppm to δ 65.5 ppm), and the neighboring carbon atom (C5″) was shifted to a higher magnetic field, about 3.1 ppm (from δ 75.1 ppm to δ 72.0 ppm), indicating the acylation took place at the C6″ atom, and the lipase of Novozym 435 showed an outstanding selectivity to the hydroxyl group at the C6″ position. The product was proved to be Man-DLD-Chol. Man-DLD-Chol was synthesized from mannose and DLD-Chol in a non-aqueous phase with the lipase of Novozym 435.

### 2.2. Characterization of Liposomes

**Figure 1:**
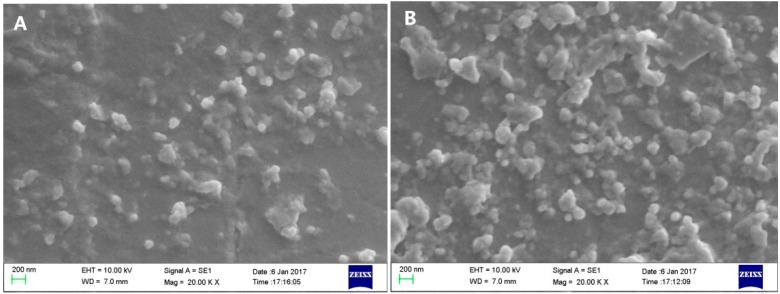
SEM images of GA liposomes (GA-Lp) (**A**) and liposomes containing mannose-diester lauric diacid-cholesterol (Man-DLD-Chol-GA-Lp) (**B**).

Scanning electron microscopy (SEM) images of GA-Lp and Man-DLD-Chol-GA-Lp are shown in Figure 1. The mean particle sizes of liposomes (GA-Lp and Man-DLD-Chol-GA-Lp) are less than 150 nm with a polydispersity index about 0.14 (Table 1). The particle size of Man-DLD-Chol-GA-Lp is slightly larger than that of GA-Lp because of Man-DLD-Chol, in which the DLD-Chol fraction of molecule was inserted in the lipid bilayer and the mannose fraction was floated on the surface of the liposomes. The zeta potential of GA-Lp and Man-DLD-Chol-GA-Lp are −32.47 ± 1.78 mV and −38.63 ± 1.07 mV (Table 1), respectively. The existence of Man-DLD-Chol might be responsible for the increased zeta potential value of Man-DLD-Chol-GA-Lp, which was beneficial for stability in the liposomes. The particle size plays an important role in drug distribution in vivo. It has been reported that the particle size of the liposomes was larger than 400 nm, which was rapidly captured by the reticuloendothelial system within minutes [46,47]. In addition, liposomes ranging from 100–200 nm in diameter were significantly accumulated in the tissue on account of permeability improvement and retention effect [48]. The encapsulation efficient (EE) of both liposomes was above 85% (Table 1). The result indicated that the incorporation of Man-DLD-Chol did not affect the EE of GA liposomes and destroy the structure of the liposomes.

### 2.3. Hemolytic Study

**Figure 2:**
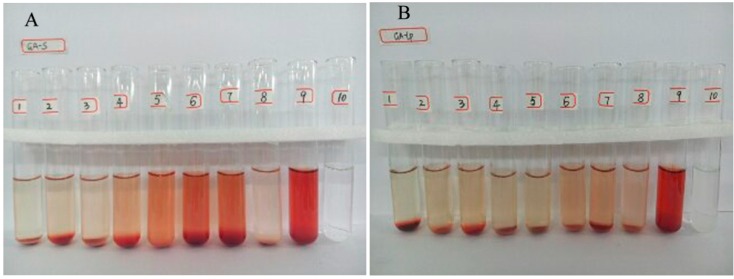

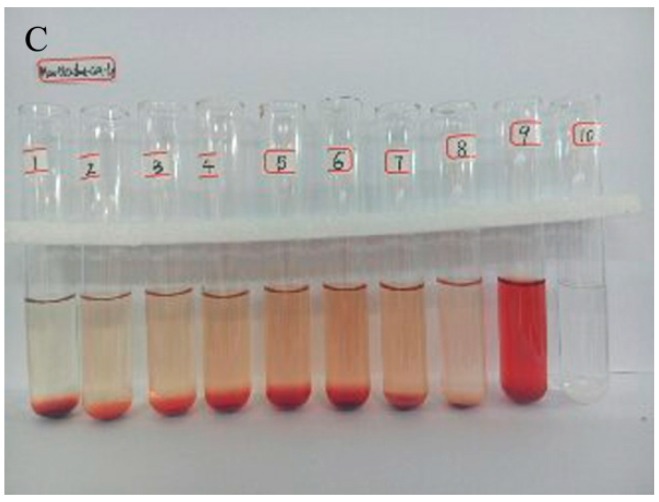
Hemolysis tests of GA-S, GA-Lp, and Man-DLD-Chol-GA-Lp ((**A**) GA-S; (**B**) GA-Lp; and (**C**) Man-DLD-Chol-GA-Lp).

**Figure 3:**
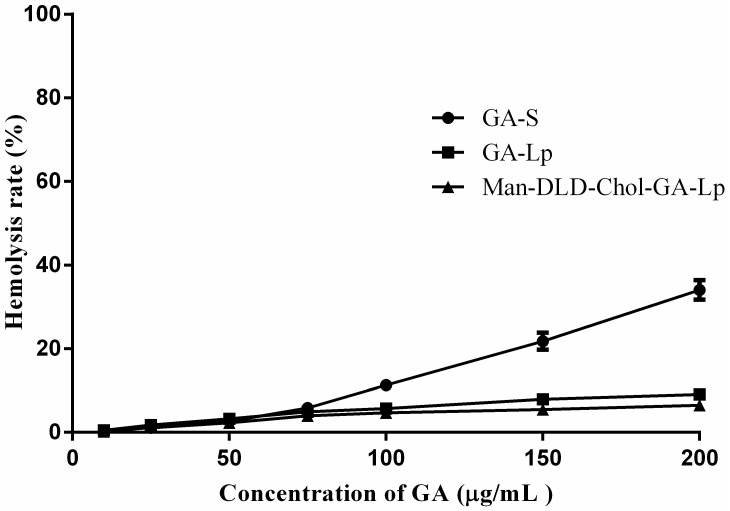
Hemolysis rates of GA solution (GA-S), GA-Lp and Man-DLD-Chol-GA-Lp.

Hemolysis data is shown in Figure 2. For the groups of GA-Lp (Figure 2B) and Man-DLD-Chol-GA-Lp (Figure 2C), we observed that hemolysis of erythrocyte suspensions only occurred obviously in the positive control (the ninthtube). However, erythrocyte suspensions containing GA-S (the fifth through seventh tubes) and the positive control (the ninthtube) appeared in different degrees of hemolysis. In addition, the data of the hemolytic rate is shown in Figure 3. Hemolytic rates of Man-DLD-Chol-GA-Lp and GA-Lp were less than 10% at the maximum GA concentration (200 μg/mL), but the hemolytic rate reached up to 34% in GA-S. These hemolytic results suggest that GA liposome would be a safe formulation for injection; meanwhile, Man-DLD-Chol was a safe drug carrier for targeted drug delivery.

### 2.4. Drug Release of Liposome In Vitro

**Figure 4:**
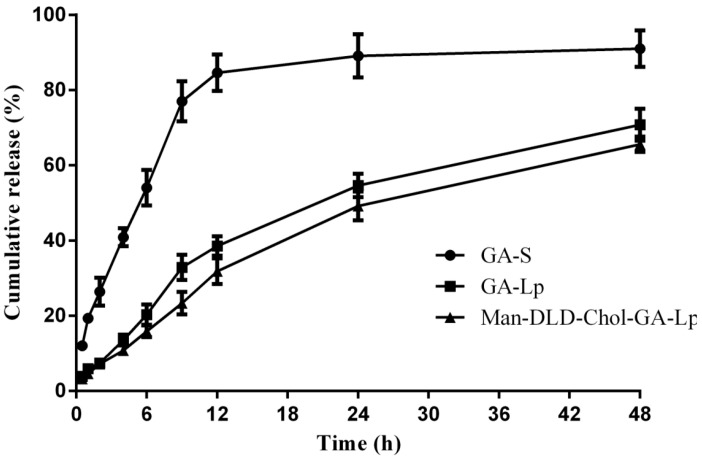
In vitro cumulative release of GA-S, GA-Lp and Man-DLD-Chol-GA-Lp.

The GA release profile from GA-S, GA-Lp and Man-DLD-Chol-GA-Lp is shown in Figure 4. GA-Lp and Man-DLD-Chol-GA-Lp were released more slowly than GA-S. The total amount of GA released from GA-S was approximately 91% in 48 h. However, we found that nearly 31% of the total drug was released from Man-DLD-Chol-GA-Lp in 12 h, and then followed by the release of 65% within 48 h. The result indicated that GA could be released slowly from the liposomes. In addition, there was no significant difference between the GA release from Man-DLD-Chol-GA-Lp and GA-Lp under the same condition. This result also suggested that the Man-DLD-Chol did not affect the structure of liposomes. To further evaluate the rule of liposomes’ release in vitro, the release data was calculated by using different formula models. The correlation coefficients in different model are listed in Table 2. It was demonstrated that the release feature of GA-S was fitted for the first-order formula model because of correlation coefficient −0.992, while the GA-Lp and Man-DLD-Chol-GA-Lp were suited to the Higuchi formula model. These results indicated that GA-S was released at a constant rate, which was related to the time. However, the GA-Lp and Man-DLD-Chol-GA-Lp were released at a non-constant rate, and the rate of drug releases was slower.

### 2.5. Cellular Uptakes

**Figure 5:**
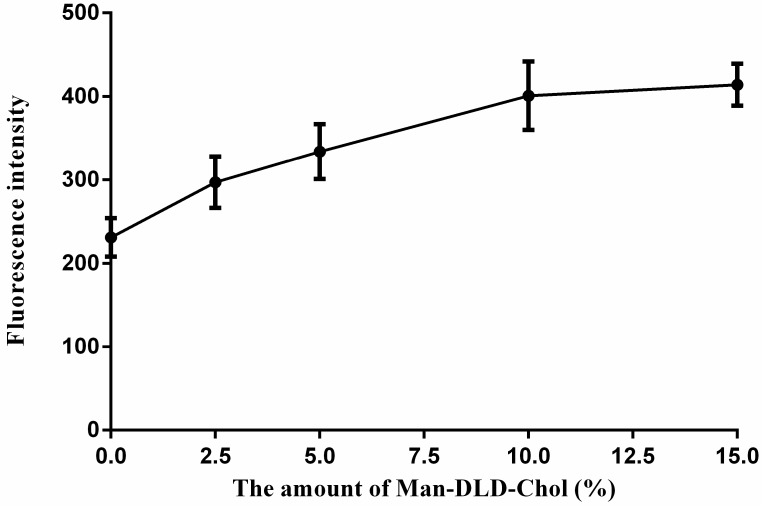
In vitro cellular uptake of Man-DLD-Chol-C_6_-Lp.

**Figure 6:**
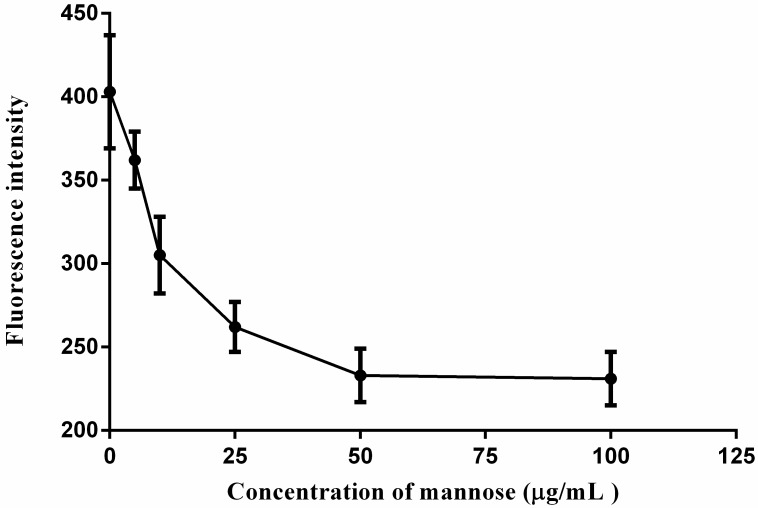
In vitro cellular uptake inhibitions by mannose.

The cellular uptakes are shown in Figure 5, the fluorescence intensity in cells incubated with Man-DLD-Chol-C_6_-Lp was significantly higher than that in C_6_-Lp. In addition, the fluorescence intensity was enhanced as the amount of Man-DLD-Chol increased. As shown in Figure 6; meanwhile, the HepG2 cells’ association of Man-DLD-Chol-C_6_-Lp (10% Man-DLD-Chol) was inhibited by pre-incubation with mannose. We found that the fluorescence decreased rapidly as the concentration of mannose increased. This result indicated that the Man-DLD-Chol lost the superior targeting effect on HepG2 cells since mannose previously inhibited mannose receptors to recognize Man-DLD-Chol. Therefore, Man-DLD-Chol could enhance cell localization and interaction of liposomes by a specific receptor-endocytosis mechanism in HepG2 cells.GA-Lp and Man-DLD-Chol-GA-Lp might be transported into HepG2 cells by non-specific and receptor-mediated endocytosis, respectively, and then exhibited the different target effect after being released from the carrier.

### 2.6. Pharmacokinetics Study

**Figure 7:**
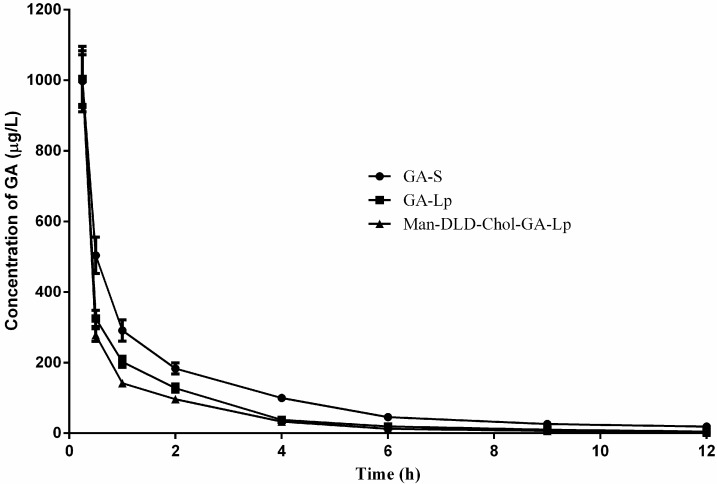
Mean plasma concentration of GA in rabbit after intravenous administration (mean ± SD; *n* = 6).

Based on the cellular uptake of liposomes mediated with Man-DLD-Chol in vitro, the pharmacokinetic properties of GA-S, GA-Lp, and Man-DLD-Chol-GA-Lp were studied by the detection of the GA content in rabbit plasma. The mean plasma concentration-time curves of GA after intravenous administration of GA-S, GA-Lp, and Man-DLD-Chol-GA-Lp are shown in Figure 7. Compared with GA-S and GA-Lp, the plasma concentration of GA shows a steep drop after intravenous injection of Man-DLD-Chol-GA-Lp. A non-compartment model was suitable to evaluate the plasma drug concentration time curves obtained in rabbits on the basis of the analysis of models and parameters. The main pharmacokinetic parameters are summarized in Table 3. Compared with GA-S, the elimination half-life (t_1/2z_) of GA-Lp and Man-DLD-Chol-GA-Lp decreased, whose values were 2.51 ± 0.44 h and 1.78 ± 0.08 h, respectively. The results indicated that the GA-Lp could rapidly distribute to tissue from blood, which agreed with the theory of the reticuloendothelial system. GA-Lp was swallowed up by the reticuloendothelial system, then the immune function was active, and the drug may distribute to some organs that are rich in reticular endothelia. The elimination rate of Man-DLD-Chol-GA-Lp was the fastest, indicating that Man-DLD-Chol-GA-LP might be recognized rapidly by mannose receptors. Meanwhile, the mean plasma clearance (CL) of Man-DD-Chol-GA-Lp (5.81 ± 0.30 L/(h·kg)) was significantly higher than that of GA-Lp (5.00 ± 0.30 L/(h·kg)) and GA-S (3.36 ± 0.11 L/(h·kg)). Moreover, the mean residence time (MRT_0-∞_) of Man-DLD-Chol-GA-Lp was the shortest (1.35 ± 0.05 h). These results indicated that Man-DLD-Chol-GA-Lp was eliminated more rapidly than GA-Lp and GA-S from the blood circulation system. In addition, the relative value of distribution volume (V_d_), GA-Lp (18.15 ± 3.42 L/kg) and Man-DLD-Chol-GA-LP (14.90 ± 0.55 L/kg), compared with GA-S (12.83 ± 0.88 L/kg), suggesting GA liposomes were easily distributed into tissue, which is beneficial to improving the therapeutic target effect. The area under the curve of drug concentration (AUC_0–∞_) of Man-DLD-Chol-GA-Lp was about 1.16 times less than that of GA-Lp (1053.55 ± 65.44 μg/L·h). The AUC of GA-Lp and Man-DLD-Chol-GA-Lp declined with a rising value of CL, which indicated that there was a rapid removal of drug in plasma. These results demonstrated that liposomes modified with Man-DLD-Chol had significant effects on the pharmacokinetics in comparison of GA-Lp.

### 2.7. Tissue Distribution Study

**Figure 8:**
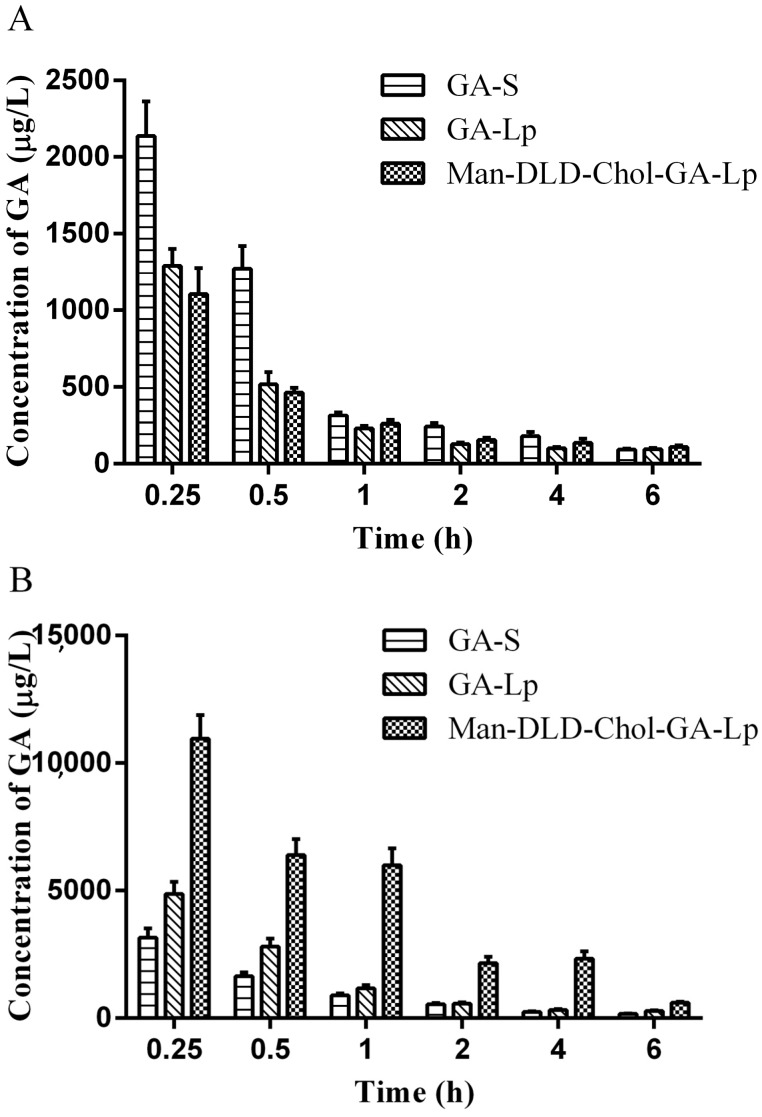

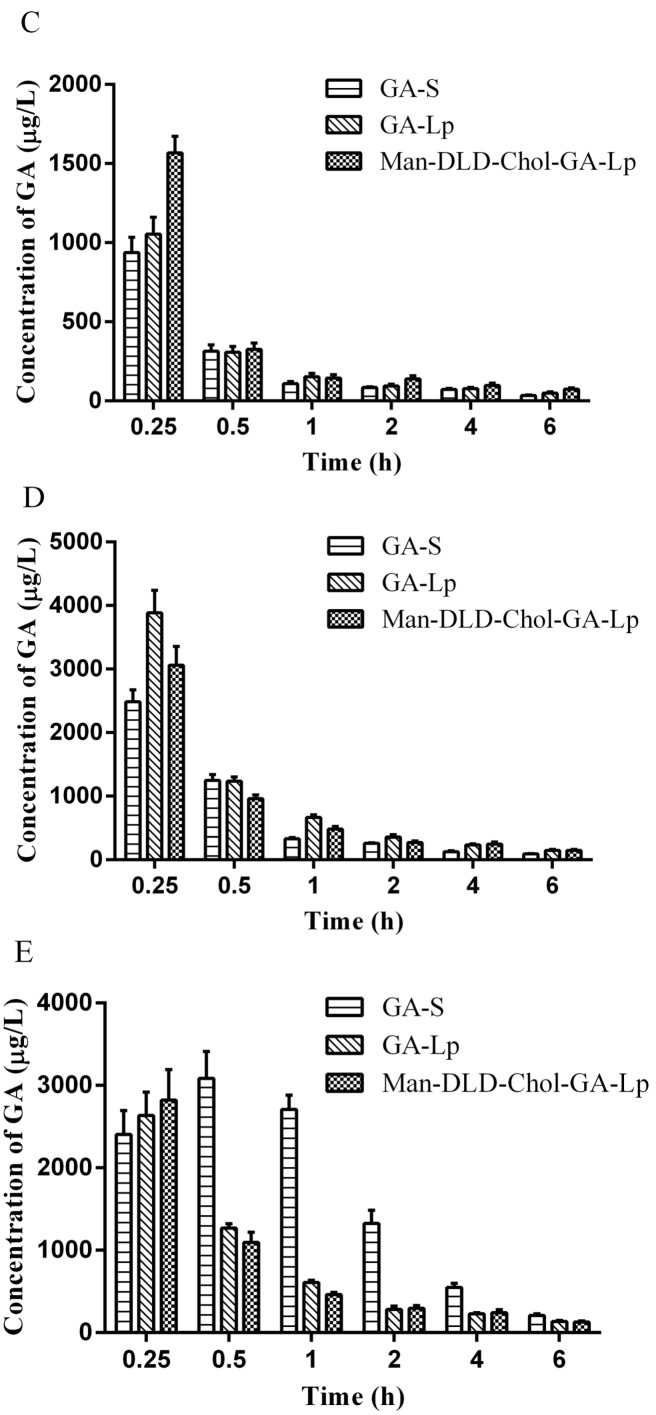

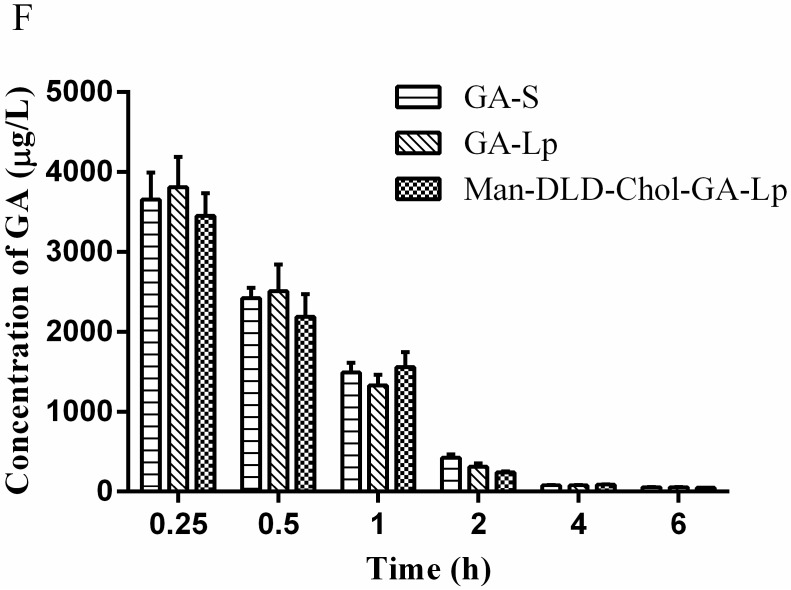
Concentration of GA in various tissues and plasma of mice after intravenous administration (*n* = 5). (**A**) Heart; (**B**)liver; (**C**) spleen; (**D**) lung; (**E**) kidney; and (**F**) plasma.

A further study of tissue distributions was needed to confirm whether the drug of Man-DLD-Chol-GA-Lp was rapidly gathered in specific organs, or not, in vivo. The linearity of the LC-MS/MS method for the detection of GA was established. Linear curves, linear coefficients, and linear ranges of GA in plasma and tissues are listed in Table 4. The concentrations of GA in heart, liver, spleen, lung, kidney, and plasma of mice were determined at various time points after intravenous administration of GA-S, GA-Lp, and Man-DLD-Chol-GA-Lp. The concentration-time profiles in various tissues and plasma are shown in Figure 8. This reflects the distribution trend of different GA formulations in vivo for mice. We found that the liver concentration of Man-DLD-Chol-GA-Lp was significantly higher than that of the other two. The result indicates that the liposome mediated with Man-DLD-Chol can deliver the drug rapidly to the liver after intravenous administration and supports our hypothesis that liposomes modified with mannosylated lipid would enhance liver-targeting through the mannose receptor. 

Furthermore, the concentration-time data was quantitatively analyzed to define the liver-targeting. Pharmacokinetic parameters of AUC_0–∞_ and C_max_ in various tissues and plasma are summarized in Table 5. Then the important parameters for the evaluation of target ability, including targeting efficiency (Te), relative targeting efficiency (R_Te_), relative uptake rate (Re), and peak concentration ratio (Ce), were calculated according to the important pharmacokinetic parameters of AUC_0–∞_ and C_max_. The equations of Te, R_Te_, Re, and Ce are reported in the Pharmacopoeia of the People’s Republic of China 2015 [49]. The data is listed in Table 6 and Table 7. Te reflects the selective rate in the targeted tissue. There was a positive correlation between the value of Te and the targeted ability. The Te of GA-S in the plasma and kidney were 30.27% and 29.14%, respectively, demonstrating the highest selection rate in the plasma and kidney. Compared with GA-S, the Te of GA-Lp in tissues was different; the high selective rate transferred into the liver and lung. The result was consistent with the theory of the reticuloendothelial system, that liposomes could be rich in the liver and lung, or might decrease the renal toxicity and side effects. With the liposome modified with Man-DLD-Chol, the Te reached 54.67% to reflect the highest selective rate in the liver, indicating that Man-DLD-Chol-GA-Lp was specifically recognized by mannose receptors; thus, the major drug was rapidly localized in the liver. In addition, R_Te_ reflects the multiple of the targeted enhancement in the liposomal formulation compared to the solution formulation. Man-DLD-Chol-GA-Lp possessed outstanding liver-targeting with an R_Te_ of 3.39 in comparison with GA-S. Re indicates the targeted ability of liposomal formulation. The Re of Man-DLD-Chol-GA-Lp was 3.34 times higher than that of GA-Lp, indicating that Man-DLD-Chol-GA-Lp had a great liver-targeting ability. A similar result regarding the Ce of Man-DLD-Chol-GA-Lp was in accordance with Te and Re, which illustrated that Man-DLD-Chol was more optimally recognized by the liver. Therefore, compared with GA-S and GA-Lp, the tissue distributions proved that Man-DLD-Chol-GA-Lp could enhance its bioavailability and targeted to the liver through mannose receptors.

## 3. Materials and Methods

### 3.1. Materials

d-(+)-Mannose (assay 97%), cholesterol and dodecanedioic acid were purchased from Aladdin Industrial Corporation (Shanghai, China). GA (assay 97.0%) was supplied by China Resources Sanjiu Medical and Pharmaceutical Co., Ltd. (Shanghai, China). Ursolic acid (assay 99.2%, the internal standard, IS) was purchased from the National Institutes for Food and Drug Control (NIFDC) (Guangzhou, China). Coumarin-6 was obtained from Beijing Norzer Pharmaceutical Co., Ltd. (Beijing, China). Egg phosphatidylcholine (EPC, assay 98%) was purchased from Lipoid Co., Ltd. (Ludwigshafen, Germany). Cholesterol (CHS, assay 98%) was purchased from Advanced Vehicle Technology Pharmaceutical, Co., Ltd. (Shanghai, China). *Candida rugosa* lipase (CRL) and molecular sieves (4Å) were purchased from Aladdin Industrial Corporation (Shanghai, China). Sephadex G-50 was purchased from Amersham Pharmacia Biotech (Piscataway, NJ, USA). Sebacic acid (assay 99%) and Novozym 435 Lipase (CAL-B) were purchased from Novozymes Biotechnology Co., Ltd. (Tianjin, China). Acetic acid vinyl ester, isooctane, pyridine, tetrahydrofuran, and other reagents were purchased from Guangzhou Chemical Reagent Factory (Guangzhou, China). Methanol and acetonitrile (HPLC grade) were obtained from Oceanpak Alexative Chemical., Ltd (Goteborg, Sweden). HPLC or LC-MC/MS reagents were filtered through a 0.22 μm filter before analysis. Methanol, ethanol, ethyl acetate, and the other reagents in liquid-liquid extraction were of analytical grade and used without further purification. Distilled water was used in all experiments.

GA-S: Sodium hydroxide (0.1 mol/L) was diluted with saline to adjust to a pH = 8.2, then 10 mg of GA was dissolved in sodium hydroxide solution (pH = 8.2) to prepare the desired concentration.

### 3.2. Synthesis of Man-DLD-Chol

**Scheme 1:**
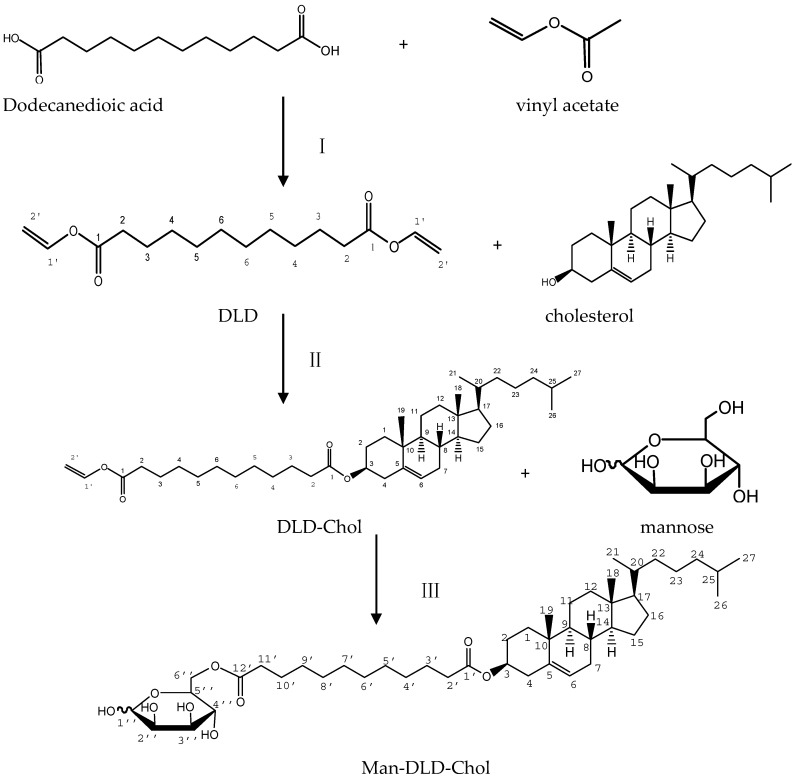
Reagents and conditions: I dodecanedioic acid, vinyl acetate, mercuric acetate, and copper acetate were combined. with magnetic stirring, for 30 min at 0 °C, then sulfuric acid was added, with magnetic stirring 9 h; II diester lauric diacid (DLD), cholesterol, isooctane, *Candida rugosa* lipase, and shaken at 250 rpm for 18 h at 46 °C; III DLD-Chol, mannose, Novozym 435 lipase, pyridine, and tetrahydrofuran were combined and shaken at 58 °C, at 250 rpm, for 27 h.

The synthetic reaction of Man-DLD-Chol was followed via three steps. For the first step, synthesis of DLD (Scheme 1-I) was synthesized. Dodecanedioic acid (17.84 g), mercuric acetate (0.50 g), copper acetate (0.10 g), and vinyl acetate (30.00 mL) were added into a round-bottom flask (250 mL) under magnetic stirring for 30 min at 0 °C. Then sulfuric acid (0.06 mL) was added. The temperature of the reaction mixtures was converted at 56 °C and the reaction time was 9 h. DLD was separated from the synthetic liquid by the method of silica gel column chromatography. In the second step, DLD-Chol (Scheme 1-II) was catalyzed by lipase, cholesterol, DLD (1:5, molar ratio), and isooctane (30 mL), which were added into a 100 mL conical vial. The reaction mixtures were shaken for 30 min in a thermostat oscillator and then *Candida rugosa* lipase (80.0 mg) was added into the vial. The reaction mixtures were shaken at 250 rpm for 18 h (46°C). DLD-Chol was obtained using ethanol recrystallization. For the final step, the synthesis of Man-DLD-Chol (Scheme 1-III) was synthesized in non-aqueous medium. Mannose, DLD–Chol (1:3.5, molar ratio), Novozym 435 (56.3 mg), pyridine, andtetrahydrofuran (2:3, volume ratio) were added into a 25 mL conical vial. The vial was placed in a thermostat oscillator at 58 °C and the reaction mixtures were shaken at 250 rpm for 27 h. The purified DLD, DLD-Chol, and Man-DLD-Chol were analyzed by MS and NMR spectroscopy. The data is shown below:

DLD: ESI-MS *m*/*z*: 305.17 [M + Na]^+^. ^13^C NMR (100 MHz, (CD_3_)_2_SO) δ: 98.3 (C1), 141.7 (C2), 170.8 (C3), 33.5 (C4), 24.5 (C5), 29.2 (C6), 29.1 (C7), 28.8 (C8). ^1^H NMR (400 MHz, (CD_3_)_2_SO) δ: 7.22 (1H, dd, *J* = 14.0, 3.6 Hz, H2), 4.76 (2H, dd, *J* = 9.9, 10.1Hz, H1), 2.41 (2H, t, *J* = 7.4 Hz, H4), 1.54 (2H, dd, *J* = 14.2, 7.1 Hz, H5), 1.25 (6H, m, H6, H7, H8).

DLD-Chol: ESI-MS *m*/*z*: 647.50 [M + Na]^+^. ^13^C NMR (100 MHz, CDCl_3_) δ: 36.8 (C1), 28.9 (C2), 73.5 (C3), 39.6 (C4), 139.5 (C5), 122.4 (C6), 31.7 (C7), 31.7 (C8), 49.9 (C9), 36.4 (C10), 20.9 (C11), 39.3 (C12), 42.1 (C13), 56.5 (C14), 24.9 (C15), 28.9 (C16), 56.0 (C17), 11.7 (C18), 19.1 (C19), 35.6 (C20), 18.5 (C21), 36.0 (C22), 23.7 (C23), 38.0 (C24), 28.1 (C25), 22.6 (C26), 22.4 (C27), 173.1 (C1′), 34.5 (C2′), 24.4 (C3′), 29.2 (C4′), 29.0 (C5′), 27.8 (C6′), 27.6 (C7′), 29.0 (C8′), 29.2 (C9′), 24.1 (C10′), 33.8 (C11′), 170.7 (C12′), 141.0 (C13′), 97.3 (C14′). ^1^H NMR (400 MHz, CDCl_3_) δ: 7.28 (1H, dd, *J* = 14.0, 6.3 Hz, H13′), 5.37 (1H, d, *J* = 4.1 Hz, H6), 4.87 (2H, dd, *J* = 14.0, 1.5 Hz, Hα14′), 4.61 (1H, m, H3), 4.56 (1H, dd, *J* = 6.5, 1.5 Hz, Hβ14′), 2.01 (2H, m, H4), 1.34 (2H, d, *J* = 2.5 Hz, H2′), 1.28 (12H, s, H4′~H9′), 1.02 (3H, s, 19-CH_3_), 0.91 (3H, d, *J* = 6.5 Hz, 21-CH_3_), 0.87 (3H, d, *J* = 1.7 Hz, 26-CH_3_), 0.87 (3H, d, *J* = 1.7 Hz, 27-CH_3_), 0.68 (3H, s, 18-CH_3_).

Man-DLD-Chol: ESI-MS *m*/*z*: 783.54 [M + Na]^+^. ^13^C NMR (100 MHz, Pry) δ: 37.4 (C1), 28.6 (C2), 74.0 (C3), 38.7 (C4), 140.1 (C5), 122.9 (C6), 32.3 (C7), 32.1 (C8), 50.4 (C9), 36.9 (C10), 21.4 (C11), 40.0 (C12), 42.6 (C13), 56.9 (C14), 24.6 (C15), 28.4 (C16), 56.5 (C17), 12.1 (C18), 19.5 (C19), 36.2 (C20), 19.1 (C21), 36.6 (C22), 24.3 (C23), 39.8 (C24), 28.3 (C25), 23.1 (C26), 22.8 (C27), 173.2 (C1′), 34.9 (C2′), 25.5 (C3′), 29.8 (C4′), 29.6 (C5′), 29.5 (C6′), 29.5 (C7′), 29.6 (C8′), 29.7 (C9′), 25.3 (C10′), 34.5 (C11′), 173.9 (C12′), 96.1 (C1″), 73.3 (C2″), 72.9 (C3″), 69.3 (C4″), 72.0 (C5″), 65.5 (C6″). ^1^H NMR (400 MHz, Pry) δ: 5.68 (1H, s, H1″), 5.17 (1H, d, *J* = 3.1 Hz, H6), 4.88 (1H, s, Hα6″), 4.66 (1H, dd, *J* = 11.0, 4.9 Hz, H3), 4.60 (1H, s, Hβ6″), 4.55 (1H, d, *J* = 3.1 Hz, H5″), 4.53 (1H, d, *J* = 2.7 Hz, H3″), 4.45 (1H, s, H2″), 4.35 (1H, t, *J* = 8.9 Hz, H4″), 2.28 (2H, m, H4), 2.18 (2H, t, *J* = 7.1 Hz, H2′), 2.05 (2H, t, *J* = 7.5 Hz, H11′), 1.47 (4H, m, H3′, H10′).

### 3.3. Liposome Preparation

The liposomes were prepared by the thin-film dispersion method [50]. In brief, lipid materials (EPC/Chol = 7/3, molar ratio), GA (8 mg), and Man-DLD-Chol (10% of EPC, molar ratio) were dissolved in chloroform, and then the organic solvent was removed to form a thin film. The lipid film was hydrated with PBS (pH = 7.4), and then the suspensions were homogenized under the ultrasonic probe. Finally, liposome suspensions were passed through a polycarbonate filter, and Man-DLD-Chol-GA-Lp was obtained. For the preparation of GA-Lp, an identical procedure was conducted except that the equivalent molar Man-DLD-Chol was replaced by cholesterol. In addition, the fluorescence blank liposomes labeled by coumarin-6 (C_6_-Lp and Man-DLD-Chol-C_6_-Lp) were also prepared by the thin-film dispersion method. The amount of coumarin-6 was 1% molar ratio of EPC.

### 3.4. Characterization of Liposome

The characteristics of liposomes, including surface morphology, particle size, zeta-potential, polydispersity index, and encapsulation efficiency, were analyzed. The surface morphology of GA-Lp and Man-DLD-Chol-GA-Lp were analyzed using SEM. The particle size and polydispersity index (PDI) of liposomes were measured by a Zetasizer Nano ZS90 (Malvern Instruments, Malvern, UK). The liposome samples were diluted with distilled water before measurement. Similarly, the zeta potential was determined by laser Doppler anemometry using a Malvern Zetasizer. Each liposome sample was measured in triplicate at 25 °C and each sample was detected for 5 min. The EE of liposomes was measured by the method reported previously [51]. The EE was determined by using gel exclusion chromatography and expressed as the ratio of liposomes containing entrapped drug and the total amount of drug in the liposome containing entrapped and non-entrapped drug. The liposome sample was passed through a Sephadex G-50 column eluted by distilled water to separate the non-entrapped drug. Then, the liposomes containing entrapped GA were disrupted with methanol to determine the concentration of GA (W_e_) by high-performance liquid chromatography (HPLC). The total amount of drug in the liposome (W_t_) containing entrapped and non-entrapped GA was also disrupted with methanol to determine the concentration of GA by HPLC.

EE (%) = W_e_/W_t_ × 100% (1)

### 3.5. Hemolytic Study

The hemolysis induced by GA-Lp and Man-DLD-Chol-GA-Lp was measured on fresh rabbit blood using the method published previously [52]. The sedimentary erythrocytes were collected, then washed with saline three times and centrifuged repeatedly until the supernatant was no longer red. Erythrocyte pellets were transferred into saline to prepare a 2% erythrocyte standard suspension. Each group were included the first through seventh tubes, and 2% erythrocyte standard suspension was incubated with different concentrations of GA-S, GA-Lp, or Man-DLD-Chol-GA-Lp; in the eighth tube, 2% erythrocyte standard suspension was incubated with 0.9% saline as a negative control; in the ninth tube, 2% erythrocyte standard suspension was incubated with the same volume of distilled water as a positive control; in the tenth tube, were references of GA-S, GA-Lp, or Man-DLD-Chol-GA-Lp. After blending, all the samples were incubated at 37 °C and observed after 6 h. In addition, all the samples were centrifuged at 8000 rpm for 10 min to separate the supernatant. The sample of erythrocyte standard suspension containing liposome (A_s_), negative control (A_0%_) and positive control (A_100%_) were determined spectrophotometrically at 540 nm. The following equation was used to calculate the hemolytic rate:

Hemolytic rate (%) = (A_s_ − A_0%_)/(A_100%_ − A_0%_) × 100% (2)

### 3.6. Drug Release from Liposome In Vitro

Release of GA from Man-DLD-Chol-GA-Lp, GA-Lp and GA-S in vitro was evaluated by the dialysis bag method [53]. GA-Lp and Man-DLD-Chol-GA-Lp containing 10 mg GA were dissolved in 4 mL PBS (pH 7.4). Both liposome suspensions and GA-S (10 mg equal to GA) were, respectively, placed in a dialysis bag (molecular weight cut of 8000–14,000). Then, the dialysis bag was suspended in 500 mL of PBS (pH 7.4) containing 0.5% Tween-80 under sinking conditions (100 rpm, 37 °C ± 0.5 °C). Samples (2 mL) were taken at predetermined time intervals from the release medium and then refilled with the same volume of fresh release medium. The samples were passed through a filter with 0.22 μm pore size. All tests were performed in triplicate. The concentration of GA was determined by HPLC. The accumulated release of GA-S, GA-Lp, and Man-DLD-Chol-GA-Lp were calculated by the following equation:

Drug release percentage (%) = W_release_/W_total_ × 100% (3)

### 3.7. Cellular Uptakes

To evaluate the cellular uptake of liposome mediated with Man-DLD-Chol in HepG2 cells, coumarin-6, as a fluorescent indicator, was labeled in the liposomes. The cellular uptake rate of fluorescent liposomes was investigated as reported previously [54]. HepG2 cells were seeded into 24-well plates at a density of 1.0 × 10^5^ cells per well and cultured at 37 °C for 24 h. Then, cells were incubated with coumarin-6 liposome (C_6_-Lp) modified with different amounts of Man-DLD-Chol (Man-DLD-Chol-C_6_-Lp) for 2 h at 37 °C. The cells were washed three times with cold PBS after the incubation, and then solubilized in 1% TritonX-100 solution. The fluorescence intensity was analyzed by a microplate reader (Molecular Devices, Waltham, MA, USA) at wavelengths of 466 nm (excitation) and 539 nm (emission). The cells without any treatment were used as the blank control. In addition, we developed a competitive binding experiment to evaluate mannose which specifically mediated the cellular uptake of mannose encapsulated in Man-DLD-Chol-C_6_-Lp. HepG2 were exposed to mannose with different concentration for 4 h beforehand, then incubated with Man-DLD-Chol-C_6_-Lp for a further 2 h. After incubation, the cells were washed three times with PBS (pH 7.4) and then were solubilized in 1% Triotnx-100 solution. The cell-associated fluorescence intensity was measured by a microplate reader.

### 3.8. Pharmacokinetic Studies

Animal experiments were performed according to the Guidelines of the Animal Center of Guangzhou University of Chinese Medicine and the Code of Practice for Housing and Care of Animals Used in Scientific Procedure (the ethic approval number of animal experiment: GDPTTCM170221). The pharmacokinetic properties of GA-S, GA-Lp and Man-DLD-Chol-GA-Lp were evaluated by the determination of the GA content in rabbit plasma. Eighteen experimental rabbits were randomly divided into three groups, including GA-S, GA-Lp, and Man-DLD-Chol-GA-Lp. Each rabbit was intravenously administered via the ear vein at a single dose of 5.25 mg/kg. Blood samples (0.5 mL) were collected from the marginal ear vein at various times (0.25, 0.5, 1, 2, 4, 6, 9, and 12 h) after administration plasma was immediately centrifuged (8000 rpm, 10 min). Two-hundred microliters (200 µL) rabbit plasma, 50 µL (500 ng/mL) ursolic acid solution (internal standard), and 2 mL of ethyl acetate were added and mixed for 5 min by vortex. After centrifugation (8000 rpm, 10 min), the clear supernatant was transferred to centrifuge tubes and dried in a vacuum oven until the organic solvent was removed. The dry sample was reconstituted with 200 µL of acetonitrile vortex-mixed, and centrifuged (10,000 rpm, 8min). Then 5 µL of the clear supernatant was injected into LC-MS/MS, and the parameters were determined by DAS2.0 software.

LC-MS/MS system condition: A BDS HYPERSIL C18 column (5 mm, 50 mm × 2.1 mm, Thermo Scientific, Boston, MA, USA) was used for separation. The mobile phase consisting of acetonitrile-5 mmol ammonium acetate solvent (70:30, *v*/*v*) was chosen. The flow rate was 0.3 mL/min and the total run time was 5 min. Mass spectrometry was conducted with a Thermo Scientific TSQ Quantum MS/MS system. It was required that the electrospray ionization (ESI) source be in negative-ion mode. The instrument parameters were set as follows: at nitrogen gas temperature (300 °C), spray voltage (3000 V), sheath gas pressure (30 psi), auxiliary gas pressure (10 psi), capillary temperature (300 °C), collision gas pressure (34 V), and the scanning time (5 min). 

### 3.9. Tissue Distributions

Animal experiments were performed according to the Guidelines of the Animal Center of Guangzhou University of Chinese Medicine and the Code of Practice for Housing and Care of Animals Used in Scientific Procedure (the ethic approval number of animal experiment: GDPTTCM170420). Mice were kept fasting overnight with free access to water before experiments. Kunming mice (18 ± 5 g) were randomly divided into three groups (GA-S, GA-Lp, and Man-DLD-Chol-GA-Lp). In the experiment, each group of mice was administered by caudal vein at a dose of 15.6 mg/kg. After injection, blood samples (0.50 mL) were obtained at various times (0.08, 0.25, 0.5, 1, 2, 4, 6, 12, 24 h). Mice were euthanized immediately and the hearts, livers, spleens, lungs, and kidneys were collected. Plasma was separated by centrifugation (12,000 rpm, 10 min, 4 °C), then stored at −20 °C until use. Tissue samples were washed and homogenized is saline and stored at −20 °C. The following steps were performed according to the description of plasma preparation in pharmaceutics. Finally, 5 µL of sample supernatant was detected by LC-MS/MS system for analysis. The parameters were measured by a non-compartmental analysis using the DAS 2.0 computer program. According to the Pharmacopoeia of the People’s Republic of China [49], major distribution parameters for the evaluation of liver targeting, including Te, R_Te_, Re, and Ce were measured. The parameters were calculated as follows:

Te (%) = AUC_target_/AUC_total_ × 100% (4)

R_Te_ = Te_liposome_/Te_solution_ (5)

Re = AUC_liposome_/AUC_solution_ (6)

Ce = (C_max_)_liposome_/(C_max_)_solution_ (7)

### 3.10. Statistical Analysis

The results were expressed as the mean ± standard deviation. Statistical comparisons between groups were made using one-way analysis of variance, and multiple comparisons were performed using the student’s t-test for independent groups, assuming equal variances within each group. A value of *p* < 0.05 was set as significance level for difference. All statistical analyses were performed using SPSS version 20 for Windows statistical software. 

## 4. Conclusions

At this stage of our work, we synthesized successfully a novel mannosylated glycolipid compound as a liposomal carrier. Man-DLD-Chol was synthesized from DLD, cholesterol, and mannose by two-step acylation under the lipase-catalytic condition. The chemical structures of DLD, DLD-Chol, and Man-DLD-Chol had been confirmed by ESI-MS and NMR. In addition, Man-DLD-Chol was successfully incorporated into the liposomes containing GA. Man-DLD-Chol-GA-Lp showed the particle size less than 150 nm with EE larger than 85% and a sustained release for 48 h in vitro. In the HepG2 cellular uptake, Man-DLD-Chol-C_6_-Lp enhanced cellular uptake and internalization of GA into HepG2 cells. The pharmacokinetic study in rabbit plasma proved that Man-DLD-Chol-GA-Lp was eliminated more rapidly than GA-Lp and GA-S. These results suggested that the GA liposomes modified with Man-DLD-Chol could be an efficient target carrier for the treatment of hepatitis and hepatocellular carcinoma. Furthermore, the tissue distributions of Man-DLD-Chol-GA-Lp was investigated, we found that the Te, R_Te_, Re, and Ce of GA on liver were higher than other tissues, demonstrating that Man-DLD-Chol had an excellent effect on liver-targeting. These results supported our hypothesis that the liposomes containing Man-DLD-Chol, as a potential drug delivery carrier, could help to improve the therapeutic effect of hepatic diseases.

## Figures and Tables

**Table 1:** The characterizations of the liposomes (mean ± SD, *n* = 3).

Sample	Particlesize(nm)	Zeta Potential (mV)	PDI	EE (%)
GA-Lp	124.37 ± 1.43	−32.47 ± 1.78	0.13 ± 0.00	86.34 ± 2.34
Man-DLD-Chol-GA-Lp	148.10 ± 0.95	−38.63 ± 1.07	0.14 ± 0.01	88.18 ± 1.31

**Table 2:** Fitting coefficient of GA-S, GA-Lp and Man-DLD-Chol-GA-Lp in different release models.

Sample	Zero-Order Formula Model	First-Order Formula Model	Higuchi Formula Model
GA-S	0.753	−0.992	0.889
GA-Lp	0.941	−0.984	0.990
Man-DLD-Chol-GA-Lp	0.964	−0.990	0.993

**Table 3:** Pharmacokinetics parameters of GA-S, GA-Lp and Man-DLD-Chol-GA-Lp in rabbit plasma (mean ± SD, *n* = 6).

Parameters	GA-S	GA-Lp	Man-DLD-Chol-GA-Lp
AUC_0–∞_ (μg/L·h)	1562.72 ± 50.45	1053.55 ± 65.44 *	906.37 ± 48.99 *
MRT_0-∞_ (h)	2.68 ± 0.12	1.77 ± 0.06 *	1.35 ± 0.05 *
t_1/2_z (h)	2.65 ± 0.25	2.51 ± 0.44	1.78 ± 0.08 *
CLz (L/(h∙kg))	3.36 ± 0.11	5.00 ± 0.30 *	5.81 ± 0.30 *
V_d_ (L/kg)	12.83 ± 0.88	18.15 ± 3.42 *	14.90 ± 0.55

*, compared with GA-S, *p* < 0.05.

**Table 4:** Linear equation, linear coefficients, and linear ranges of GA in plasma and tissues.

Biosample	Linear Curve	Linear Coefficient (r^2^)	Linear Range (ng/mL)
Plasma	*Y* = 0.0063*X* + 0.1950	0.9991	5–5000
Heart	*Y* = 0.0054*X* + 0.2093	0.9989	5–5000
Liver	*Y* = 0.0056*X* + 0.4971	0.9993	5–12,000
Spleen	*Y* = 0.0059*X* + 0.1126	0.9994	5–5000
Lung	*Y* = 0.0067*X* + 0.3202	0.9991	5–5000
Kidney	*Y* = 0.0050*X* + 0.2621	0.9990	5–5000

**Table 5:** Pharmacokinetic parameters of GA-S, GA-Lp, and Man-DLD-Chol-GA-Lp in mice’s tissues and plasma (mean ± SD, *n* = 5).

Parameters	Tissues	GA-S	GA-Lp	Man-DLD-Chol-GA-Lp
AUC_0–t_	Heart	2.5973 ± 0.1435	1.4244 ± 0.0936 *	1.4482 ± 0.1024 *
(mg/L·h)	Liver	4.0944 ± 0.2211	5.8582 ± 0.2376	19.5541 ± 0.6356 *
	Spleen	0.9401 ± 0.0744	1.0280 ± 0.068	1.3993 ± 0.1322 *
	Lung	2.6747 ± 0.1262	3.7460 ± 0.1737	3.0815 ± 0.1363
	Kidney	7.3979 ± 0.2813	3.0836 ± 0.1424 *	3.053 ± 0.2258 *
	Plasma	7.6867 ± 0.1673	7.5861 ± 0.5270	7.2320 ± 0.1950
C_max_ (mg/L)	Heart	2.1362 ± 0.2284	1.2913 ± 0.1100 *	1.1061 ± 0.1705 *
	Liver	3.1618 ± 0.355	4.8747 ± 0.4704	10.9554 ± 0.9288 *
	Spleen	0.9382 ± 0.0954	1.0545 ± 0.1058	1.5679 ± 0.1045 *
	Lung	2.4842 ± 0.1903	3.8845 ± 0.3562 *	3.0607 ± 0.2937
	Kidney	3.1537 ± 0.1802	2.6341 ± 0.2846	2.8216 ± 0.3714
	Plasma	3.6551 ± 0.3398	3.8124 ± 0.3767	3.4521 ± 0.2840

*, compared with GA-S, *p* < 0.05.

**Table 6:** Targeting parameters (Te) of GA-S in mice tissues and plasma.

Parameter	Heart	Liver	Spleen	Lung	Kidney	Plasma
Te (%)	10.23	16.13	3.70	10.53	29.14	30.27

**Table 7:** Targeting parameters of GA-Lp, and Man-DLD-Chol-GA-Lp in mice tissues and plasma.

Parameter	Formulation	Heart	Liver	Spleen	Lung	Kidney	Plasma
Te (%)	GA-Lp	6.27	25.78	4.52	16.48	13.57	33.38
	Man-DLD-Chol-GA-Lp	4.05	54.67	3.91	8.62	8.54	20.22
R_Te_	GA-Lp	0.61	1.60	1.22	1.56	0.47	1.10
	Man-DLD-Chol-GA-Lp	0.40	3.39	1.06	0.82	0.29	0.67
Re	GA-Lp	0.55	1.43	1.09	1.40	0.42	0.99
	Man-DLD-Chol-GA-Lp	0.56	4.78	1.49	1.15	0.41	0.94
Ce	GA-Lp	0.60	1.54	1.12	1.56	0.84	1.04
	Man-DLD-Chol-GA-Lp	0.52	3.46	1.67	1.23	0.89	0.94

## Supplementary Materials

Supplementary Materials (MR and NMR data of Man-DLD-Chol conjugate) are available online.
