# Supplementary material for: Glycyrrhetinic Acid Liposomes Containing Mannose-Diester Lauric Diacid-Cholesterol Conjugate Synthesized by Lipase-Catalytic Acylation for Liver-Specific Delivery
Source: Molecules. 2017 Sep 24;22(10):1598. doi: 10.3390/molecules22101598 (PMC6151824; doi:10.3390/molecules22101598)
Supplement: Supplementary file 1 [file molecules-22-01598-s001.pdf]

Supplementary information:

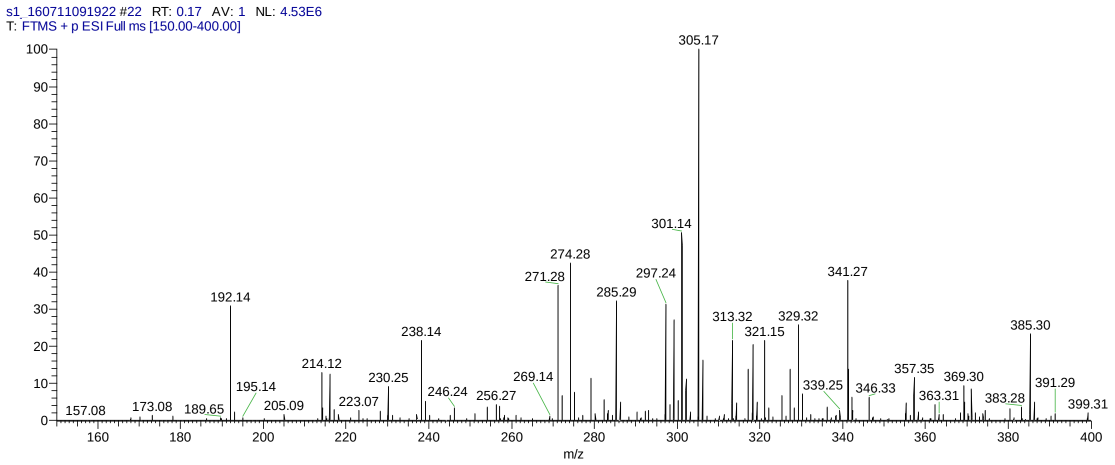

Figure S1 MS spectra a of DLD

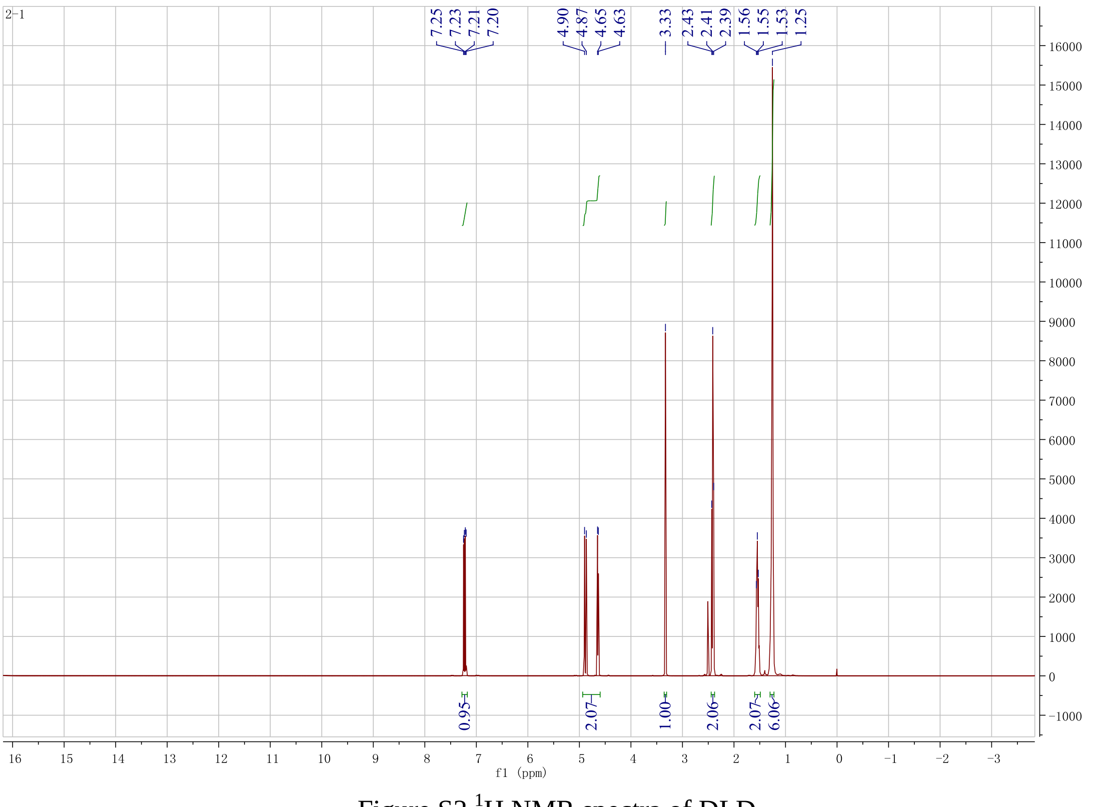

Figure S2 ^{1}H NMR spectra of DLD

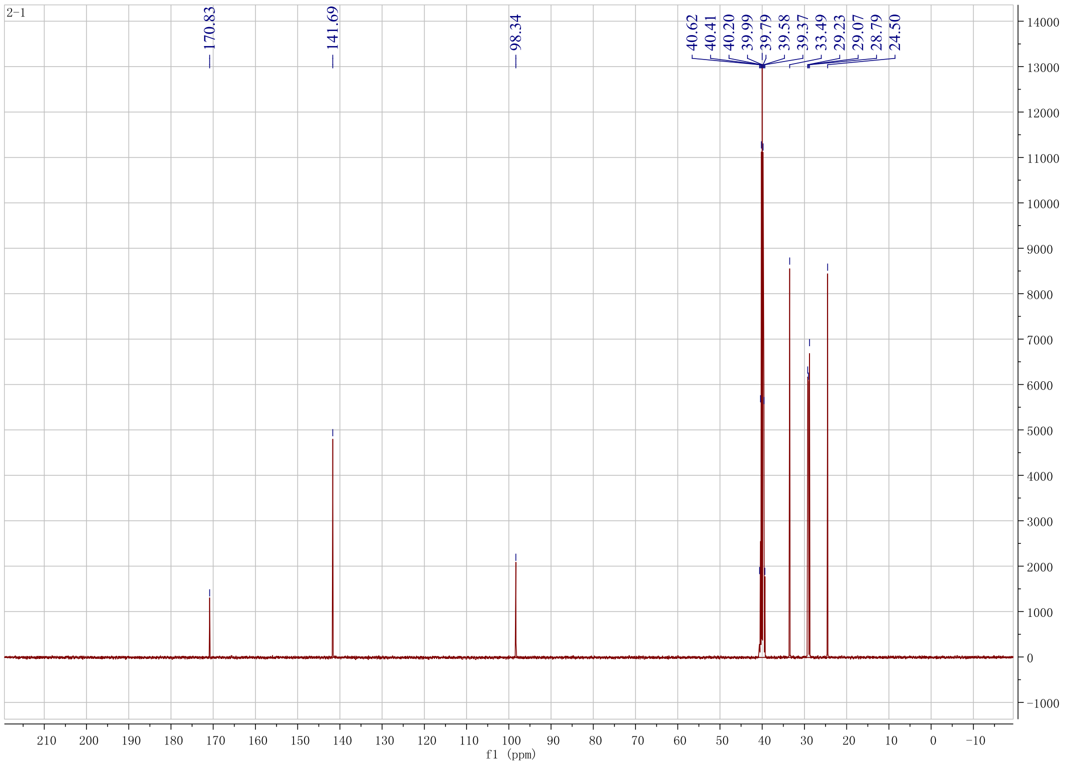

Figure S3 ^{13}C NMR spectra of DLD

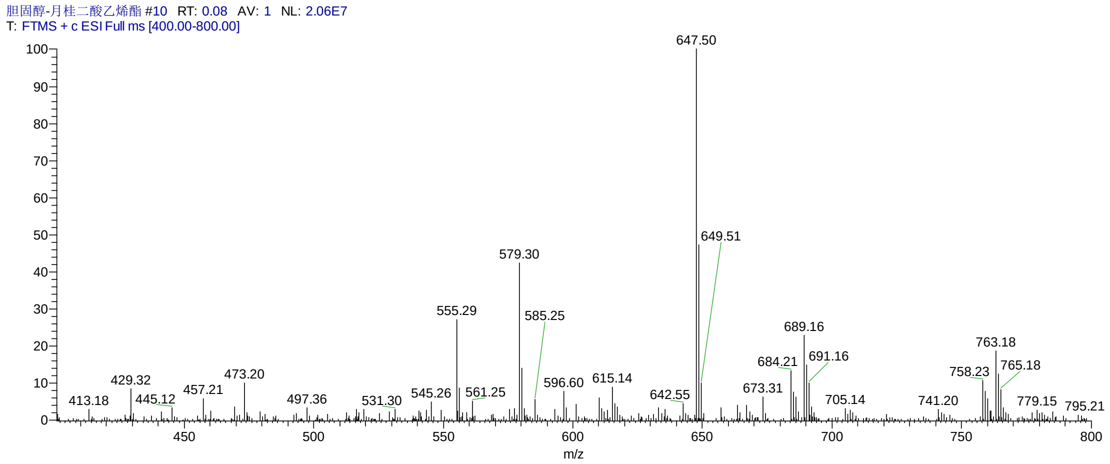

Figure S4 MS spectra of DLD-Chol

(B)

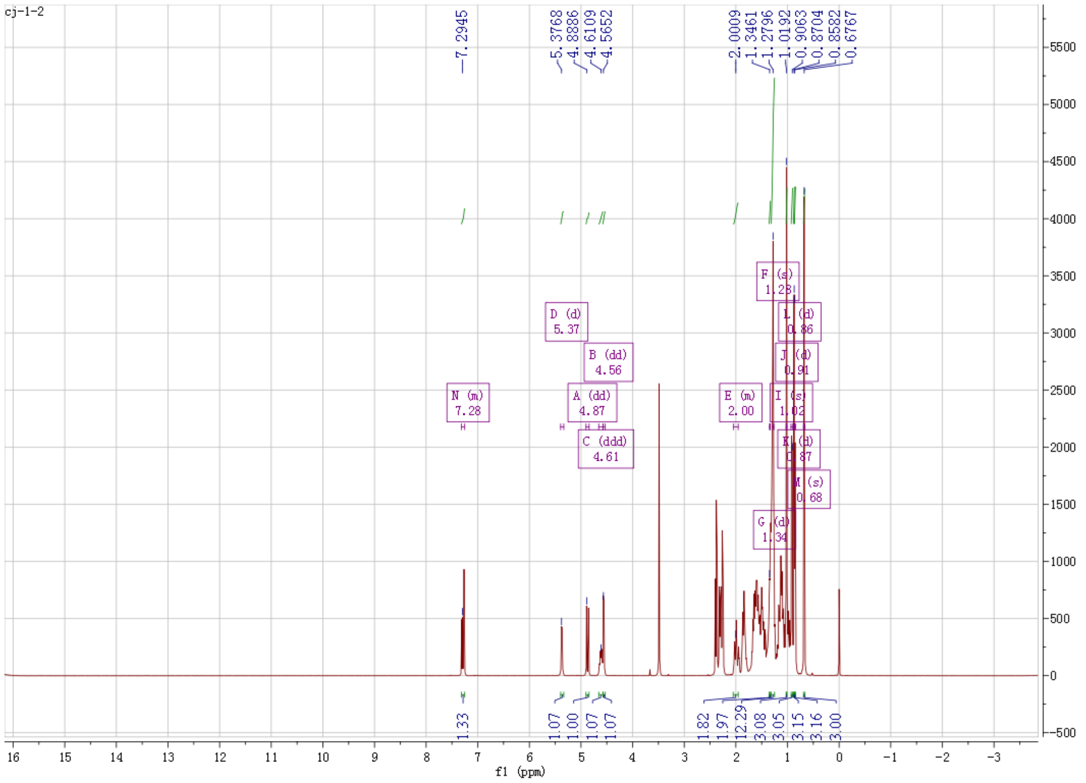

Figure S5 ^{1}H NMR spectra of DLD-Chol

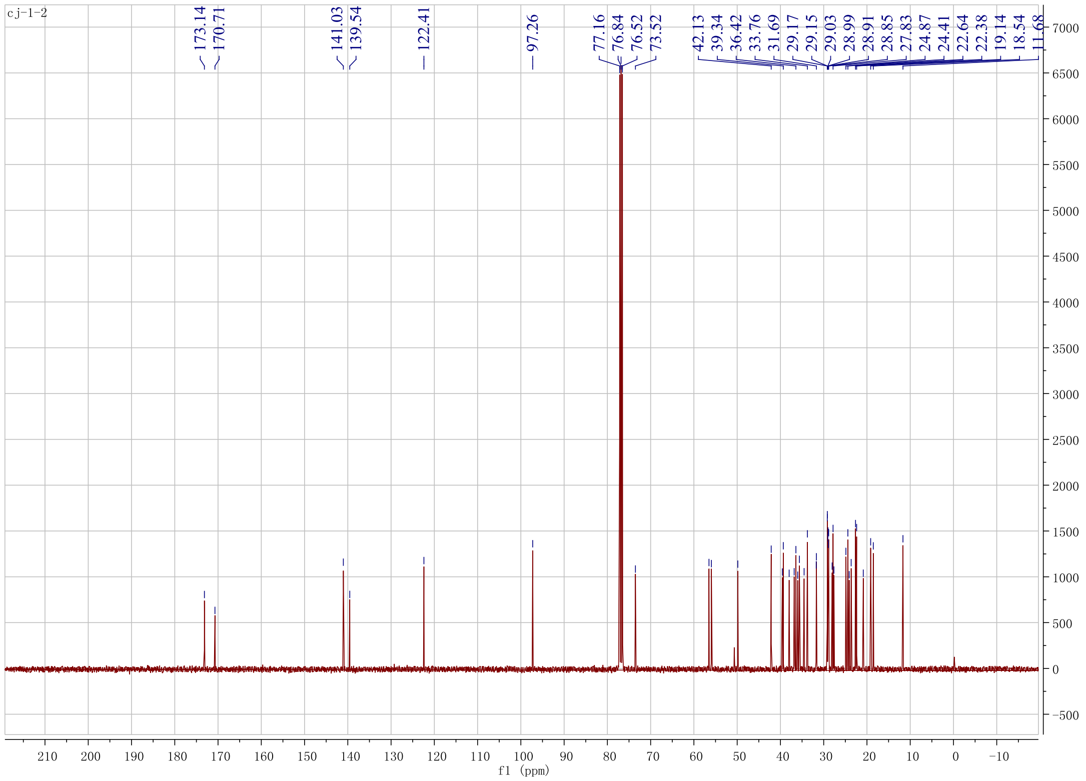

Figure S6 ^{13}C NMR spectra of DLD-Chol

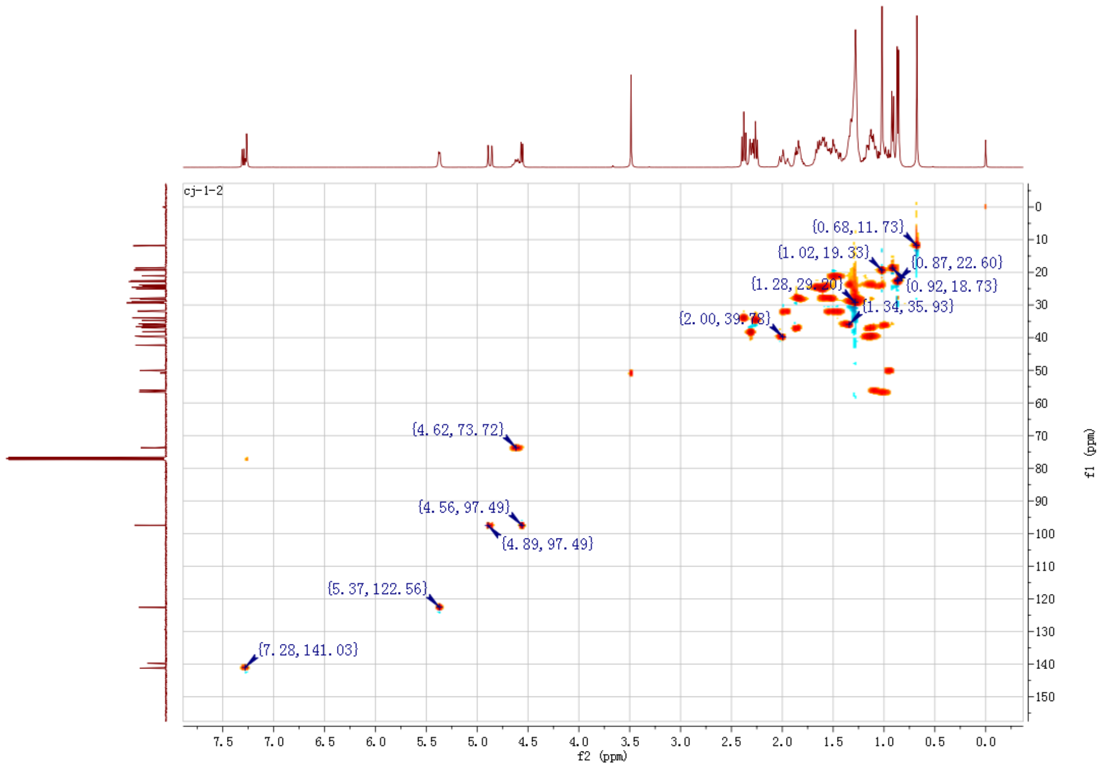

Figure S7 HMQC spectra of DLD-Chol

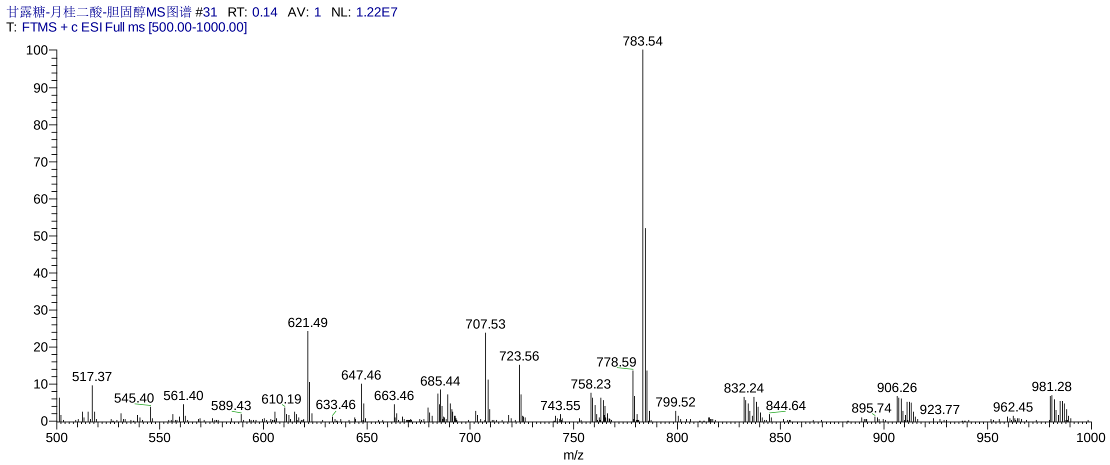

Figure S8 MS spectra of Man-DLD-Chol

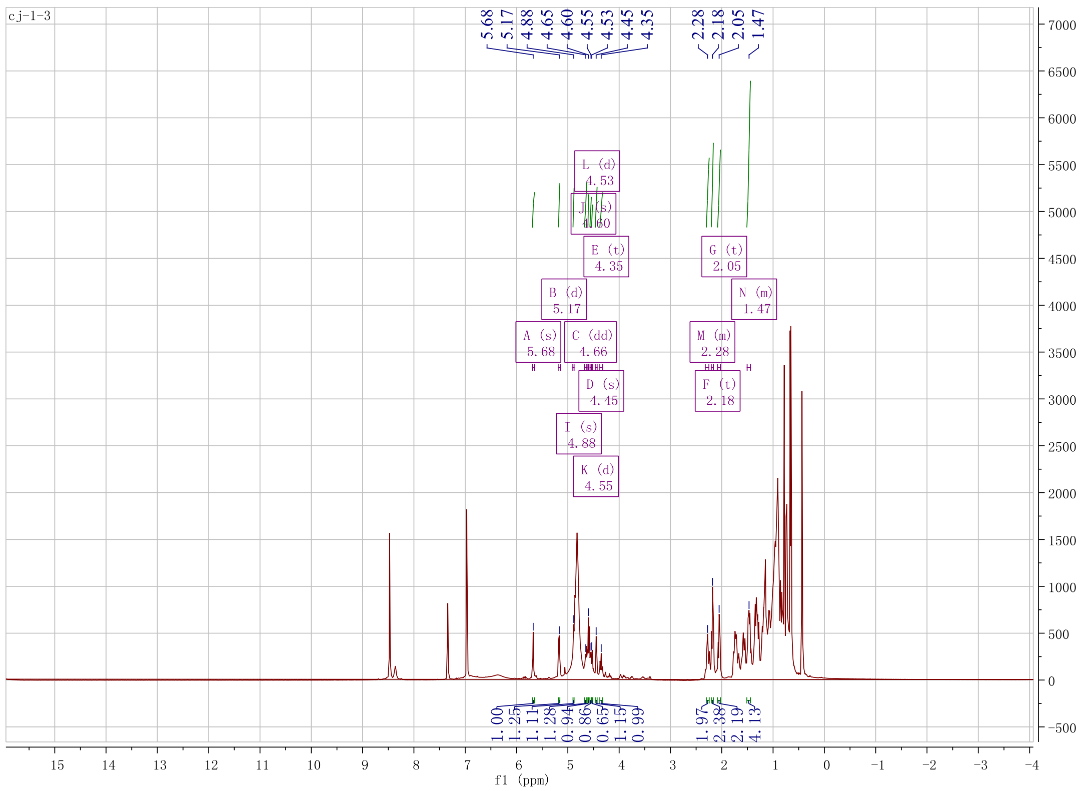

Figure S9 ^{1}H NMR spectra of Man-DLD-Chol

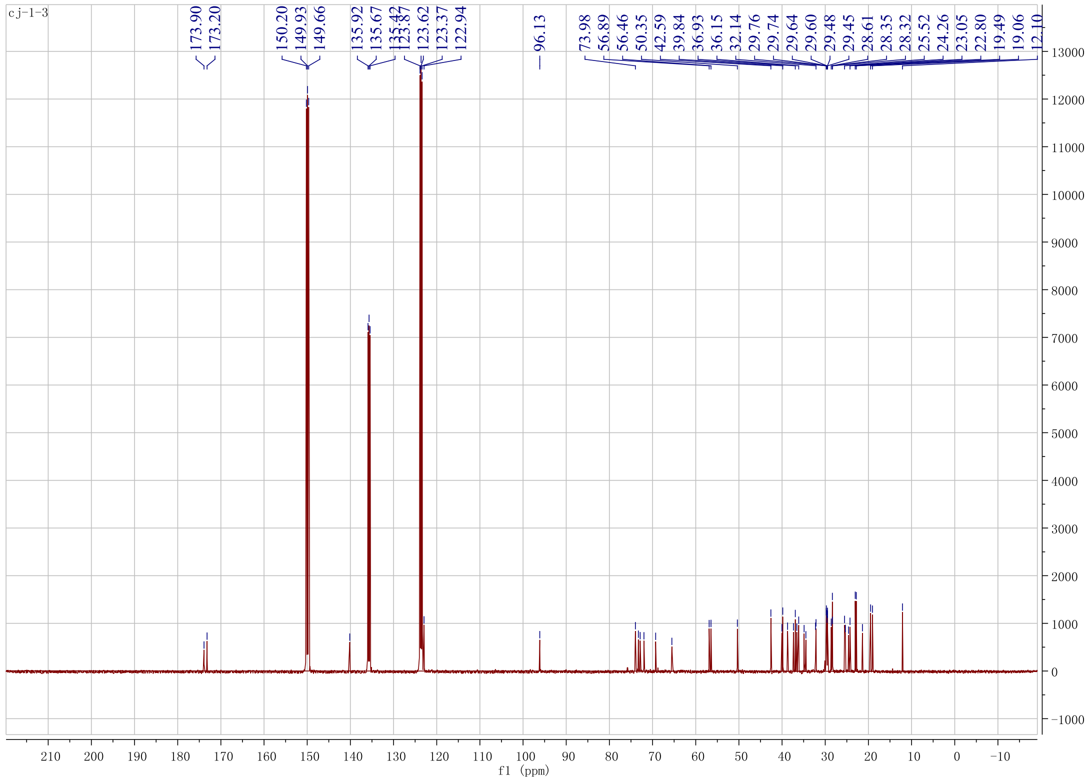

Figure S10 ^{13}C NMR spectra of Man-DLD-Chol

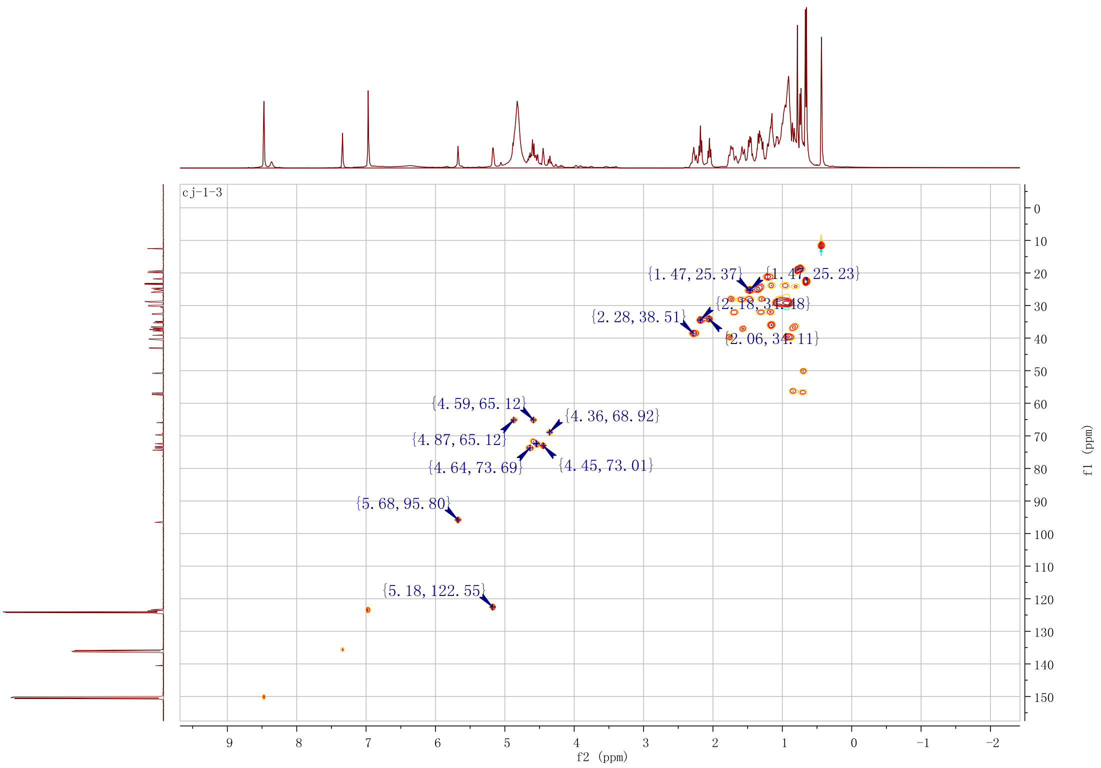

Figure S11 HMQC spectra of Man-DLD-Chol

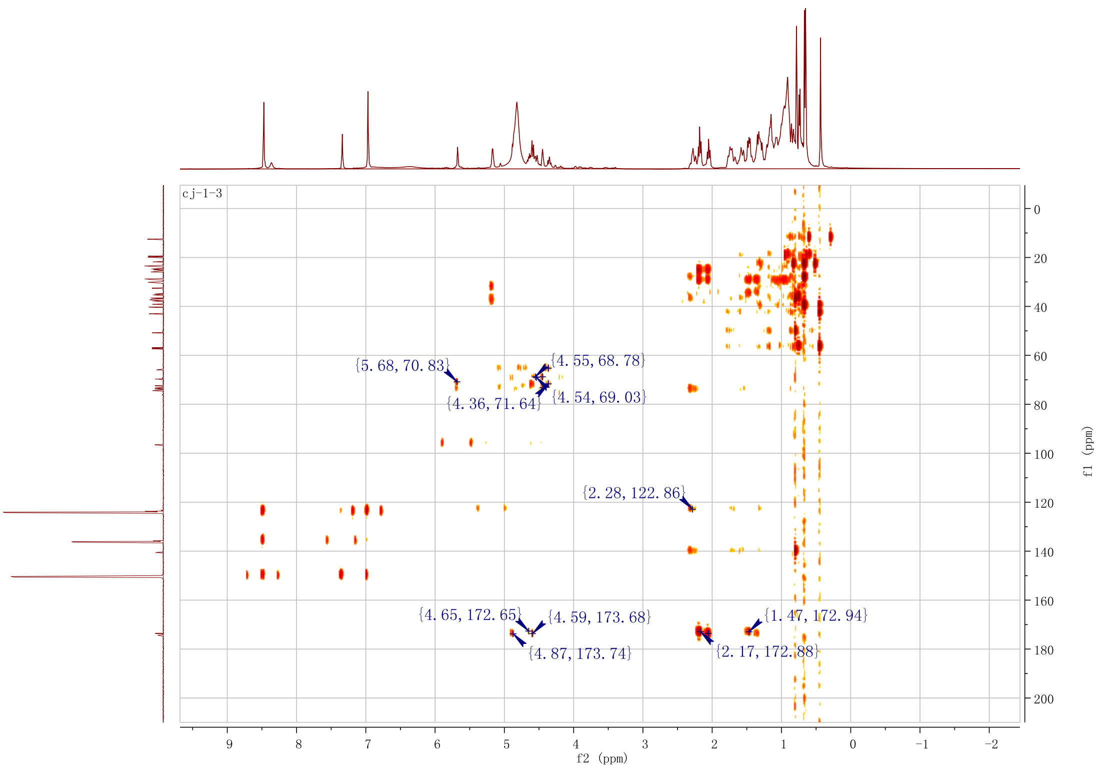

Figure S12 HMBC spectra of Man-DLD-Chol
